# Silicious Gutta Percha Pencils for Removal of Discolorations from the Teeth

**Published:** 1856-01

**Authors:** 


*Silicious Gutta Percha Pencils for Removal of Discolorations from the Teeth.*—Mr. Cyphers, a student of the Baltimore Dental College, has recently constructed a most useful and valuable instrument, for the removal of discolorations from the teeth. It consists of gutta percha and finely powdered Arkansas rock or quartz; the latter is largely incorporated in the former, which is prepared in cylindrical pieces of about a quarter of an inch in diameter, and four or five inches in length. We have used it in a number of cases, and find it better adapted for the removal of discolorations from the teeth, than anything we have ever before employed. The practical value of the thing cannot fail to be appreciated by every dentist. Accompanying each box of these gutta percha sticks, are several pieces of tape coated with the same article, filled with very finely pulverized quartz. They are designed for acting on the approximal surfaces of the teeth, for the removal either of discolorations or removing file scratches from the surfaces of fillings in these localities.

Since writing the above we have received from a dentist of Baltimore, a communication endorsing the favorable opinion above expressed, with regard to the practical value of Mr. Cyphers' silicious gutta percha pencils. He says, he finds them so admirably adapted for cleansing and polishing the teeth and for finishing all kinds of plate work that he has no doubt their use, by the profession, will soon become universal. With a view of giving dentists an opportunity of trying his silicious gutta percha pencils and ribbons, Mr. Cyphers has placed a few of them in the hands of Dr. Blandy, put up very neatly in small parcels, ranging in price from 50 cents to one dollar.

